# Ethyl 2-[(2-hy­droxy­benzyl­idene)amino]-6-methyl-4,5,6,7-tetra­hydro­thieno[2,3-*c*]pyridine-3-carboxyl­ate

**DOI:** 10.1107/S1600536813016474

**Published:** 2013-06-19

**Authors:** Naki Çolak, Dursun Ali Köse, Nazmiye Marım, Ömer Çelik, Tuncer Hökelek

**Affiliations:** a Department of Chemistry, Hitit University, 19030 Çorum, Turkey; b Department of Physics, Dicle University, 21280 Sur, Diyarbakır, Turkey; c Department of Physics, Hacettepe University, 06800 Beytepe, Ankara, Turkey

## Abstract

The title compound, C_18_H_20_N_2_O_3_S, exists as the phenol–imine form in the crystal and there are bifurcated intra­molecular O—H⋯(N/O) hydrogen bonds present. The conformation about the C=N bond is *anti* (1*E*); the C=N imine bond length is 1.287 (4) Å and the C=N—C angle is 122.5 (3)°. In the tetrahydrothienopyridine moiety, the six-membered ring has a flattened-boat conformation. In the crystal, mol­ecules are stacked nearly parallel to (110) and a weak C—H⋯π inter­action is observed. The carbonyl O atom is disordered over two positions and was refined with a fixed occupancy ratio of 0.7:0.3.

## Related literature

For investigations of tautomerism and intra­molecular hydrogen bonds in 2-hy­droxy Schiff bases in both solution and the solid state, see: Hayvalı *et al.* (2003 ▶); Pizzala *et al.* (2000 ▶); Kaitner & Pavlovic (1996 ▶). For the role of tautomerism in Schiff bases in distinguishing their photochromic and thermochromic characteristics, see: Hadjoudis (1981 ▶); Dürr (1989 ▶); Moustakali-Mavridis *et al.* (1980 ▶). For keto-amine and phenol-imine forms observed in naphthaldimine and salicylaldimine Schiff bases, see: Gavranic *et al.* (1996 ▶); Kaitner & Pavlovic (1996 ▶); Pizzala *et al.* (2000 ▶); Hökelek *et al.* (2004 ▶). For related structures, see: Hökelek *et al.* (2000 ▶, 2004 ▶);Yıldız *et al.* (1998 ▶). For puckering parameters, see: Cremer & Pople (1975 ▶).
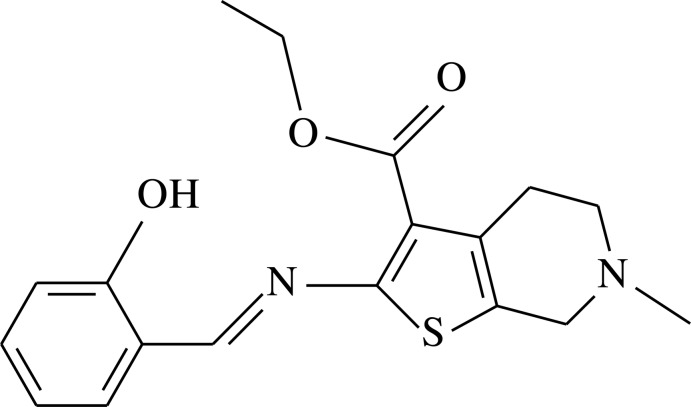


## Experimental

### 

#### Crystal data

C_18_H_20_N_2_O_3_S
*M* *_r_* = 344.42
Orthorhombic, 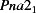

*a* = 22.0243 (5) Å
*b* = 16.1559 (4) Å
*c* = 4.8055 (1) Å
*V* = 1709.90 (7) Å^3^
*Z* = 4
Mo *K*α radiation
μ = 0.21 mm^−1^
*T* = 296 K
0.35 × 0.15 × 0.10 mm

#### Data collection

Bruker Kappa APEXII CCD area-detector diffractometer
Absorption correction: multi-scan (*SADABS*; Bruker, 2005 ▶) *T*_min_ = 0.912, *T*_max_ = 0.980
11744 measured reflections
2778 independent reflections
2120 reflections with *I* > 2σ(*I*)
*R*_int_ = 0.062

#### Refinement

*R*[*F* ^2^ > 2σ(*F* ^2^)] = 0.043
*wR*(*F* ^2^) = 0.104
*S* = 1.02
2778 reflections
236 parameters
1 restraint
H atoms treated by a mixture of independent and constrained refinement
Δρ_max_ = 0.16 e Å^−3^
Δρ_min_ = −0.16 e Å^−3^
Absolute structure: Flack (1983 ▶), 691 Friedel pairs
Flack parameter: 0.08 (11)

### 

Data collection: *APEX2* (Bruker, 2007 ▶); cell refinement: *SAINT* (Bruker, 2007 ▶); data reduction: *SAINT*; program(s) used to solve structure: *SHELXS97* (Sheldrick, 2008 ▶); program(s) used to refine structure: *SHELXL97* (Sheldrick, 2008 ▶); molecular graphics: *ORTEP-3 for Windows* (Farrugia, 2012 ▶); software used to prepare material for publication: *WinGX* (Farrugia, 2012 ▶) and *PLATON* (Spek, 2009 ▶).

## Supplementary Material

Crystal structure: contains datablock(s) I, global. DOI: 10.1107/S1600536813016474/su2610sup1.cif

Structure factors: contains datablock(s) I. DOI: 10.1107/S1600536813016474/su2610Isup2.hkl

Supplementary material file. DOI: 10.1107/S1600536813016474/su2610Isup3.cml

Additional supplementary materials:  crystallographic information; 3D view; checkCIF report

## Figures and Tables

**Table 1:** Hydrogen-bond geometry (Å, °) *Cg*1 is the centroid of ring *B* (S1/C8–C10/C14).

*D*—H⋯*A*	*D*—H	H⋯*A*	*D*⋯*A*	*D*—H⋯*A*
O1—H1⋯O3	0.90 (5)	2.40 (5)	3.053 (4)	129 (4)
O1—H1⋯N1	0.90 (5)	1.79 (5)	2.605 (4)	148 (4)
C12—H12*A*⋯*Cg*1^i^	0.97	2.77	3.701 (3)	161

Symmetry code: (i) 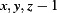.

**Fig. 1.**
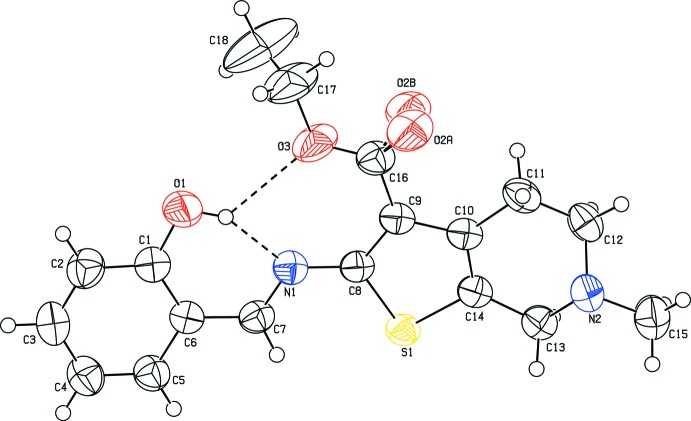
The molecular structure of the title molecule with the atom-numbering scheme. Displacement ellipsoids are drawn at the 50% probability level. Hydrogen bonds are shown as dashed lines - see Table 1 for details.

**Fig. 2.**
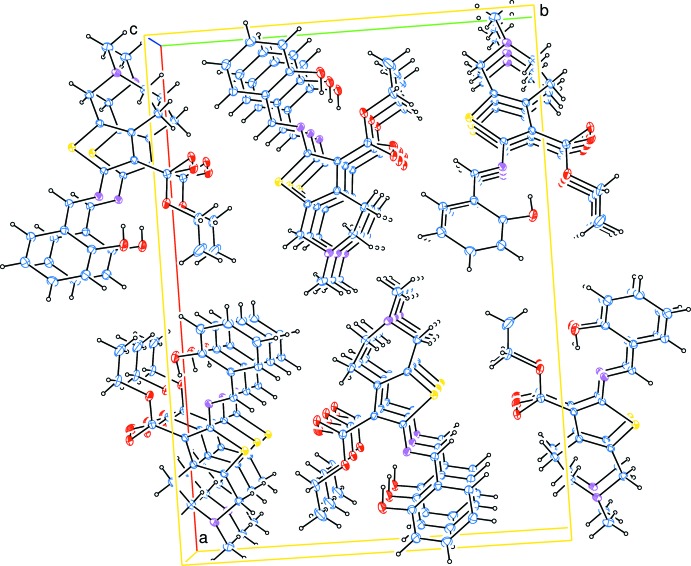
A view along the c axis of the crystal packing of the title compound.

## supplementary crystallographic information

### Comment

Tautomerism and intramolecular hydrogen bonds in 2-hydroxy Schiff bases in solution and in the solid state have been investigated using IR and UV spectroscopies (Hayvalı *et al.*, 2003), ^1^H, ^13^C and ^15^N NMR spectroscopies (Pizzala *et al.*, 2000), and X-ray crystallography techniques (Kaitner & Pavlovic, 1996). Tautomerism in Schiff bases plays an important role in distinguishing their photochromic (Hadjoudis, 1981; Dürr, 1989) and thermochromic (Moustakali-Mavridis *et al.*, 1980) characteristics. In the solid state, it is generally specified by X-ray analysis that the O···H—N (keto-amine form) is observed in naphthaldimine, while the O—H···N (phenol-imine form) is observed in salicylaldimine Schiff bases (Gavranic *et al.*, 1996; Kaitner & Pavlovic, 1996), although it is claimed that both keto-amine and phenol-imine forms are present in the crystalline state, based on NMR (Pizzala *et al.*, 2000) and X-ray studies (Hökelek *et al.*, 2004). In fact, the stereochemistry of the molecule and the type of nitrogen substituents in salicylaldimine and naphthaldimine Schiff bases are highly important on the type of hydrogen bond being observed (Hökelek *et al.*, 2004). The title 2-hydroxy Schiff base compound was synthesized and its crystal structure is reported on herein.

The molecule of the title compound is in the phenol-imine form (Fig. 1). The C═N [1.287 (4) Å] imine bond distance and C═N–C [122.5 (3)°] bond angle are comparable with the corresponding values of 1.276 (2) Å and 124.64 (17)°, and 1.279 (2) Å and 123.05 (16)° in 1,3-bis[2-(2-hydroxybenzylideneamino]phenoxy] propane, (II) (Hökelek *et al.*, 2004), 1.270 (3) Å and 123.5 (2)° in 1,8-di[N-2-oxyphenyl-salicylidene]-3,6-dioxaoctane, (III) (Yıldız *et al.*, 1998) and those of 1.288 (4) Å and 121.3 (3)°, and 1.277 (4) Å and 124.3 (3)° in 1,5-di[N-2-oxyphenyl-salicylidene]-3-oxapentane, (IV) (Hökelek *et al.*, 2000).

There are bifurcated intramolecular O—H···N and O—H···O hydrogen bonds present in the molecule (Table 1 and Fig. 1). The C6-C7═N1-C8 [178.0 (3)°] torsion angle shows that the conformation about the C═N bond is *anti* (1*E*). The planar rings *A* (C1–C6) and *B* (S1/C8–C10/C14) are oriented at a dihedral angle of 8.48 (9)°. Ring *C* (N2/C10–C14) has a flattened-boat conformation [φ = -23.8 (5)° and θ = 128.0 (4)°] having a total puckering amplitude Q_T_ of 0.500 (3) Å (Cremer & Pople, 1975).

In the crystal, molecules are stacked nearly parallel to (110) and a weak C—H···π interaction is observed (Table 1 and Fig. 2).

### Experimental

A mixture of 2-hydroxybenzaldehyde (1.22 g, 10 mmol) and ethyl 2-amino-6-methyl -4,5,6,7-tetrahydrothieno[2,3-c]pyridine-3-carboxylate (2.40 g, 10 mmol) in ethanol (20 ml) was refluxed for 2 h, and then cooled to room temperature. The precipitated solid was collected, washed with cold ethanol and recrystallized from ethanol giving orange block-like crystals.

### Refinement

Atoms H1 (for OH) and H7 (for methine) were located in a difference Fourier map and refined freely. The C-bound H atoms were positioned geometrically with C—H = 0.93, 0.96 and 0.97 Å for aromatic, methyl and methylene H atoms, respectively, and constrained to ride on their parent atoms, with *U*_iso_(H) = k × *U*_eq_(C), where k = 1.5 for methyl H-atoms and k = 1.2 for other H-atoms. During the refinement process the disordered O2A and O2B atoms were refined with a fixed occupancy ratio of 0.70:0.30.

### Crystal data

**Table:**

C_18_H_20_N_2_O_3_S	*F*(000) = 728
*M**_r_* = 344.42	*D*_x_ = 1.338 Mg m^−^^3^
Orthorhombic, *P**n**a*2_1_	Mo *K*α radiation, λ = 0.71073 Å
Hall symbol: P 2c -2n	Cell parameters from 2064 reflections
*a* = 22.0243 (5) Å	θ = 3.1–23.9°
*b* = 16.1559 (4) Å	µ = 0.21 mm^−^^1^
*c* = 4.8055 (1) Å	*T* = 296 K
*V* = 1709.90 (7) Å^3^	Block, orange
*Z* = 4	0.35 × 0.15 × 0.10 mm

### Data collection

**Table:**

Bruker Kappa APEXII CCD area-detector diffractometer	2778 independent reflections
Radiation source: fine-focus sealed tube	2120 reflections with *I* > 2σ(*I*)
Graphite monochromator	*R*_int_ = 0.062
φ and ω scans	θ_max_ = 27.0°, θ_min_ = 1.6°
Absorption correction: multi-scan (*SADABS*; Bruker, 2005)	*h* = −28→28
*T*_min_ = 0.912, *T*_max_ = 0.980	*k* = −20→20
11744 measured reflections	*l* = −5→6

### Refinement

**Table:**

Refinement on *F*^2^	Secondary atom site location: difference Fourier map
Least-squares matrix: full	Hydrogen site location: inferred from neighbouring sites
*R*[*F*^2^ > 2σ(*F*^2^)] = 0.043	H atoms treated by a mixture of independent and constrained refinement
*wR*(*F*^2^) = 0.104	*w* = 1/[σ^2^(*F*_o_^2^) + (0.0469*P*)^2^ + 0.1687*P*] where *P* = (*F*_o_^2^ + 2*F*_c_^2^)/3
*S* = 1.02	(Δ/σ)_max_ = 0.001
2778 reflections	Δρ_max_ = 0.16 e Å^−^^3^
236 parameters	Δρ_min_ = −0.16 e Å^−^^3^
1 restraint	Absolute structure: Flack (1983), 691 Friedel pairs
Primary atom site location: structure-invariant direct methods	Flack parameter: 0.08 (11)

### Special details

**Table:**

Geometry. All esds (except the esd in the dihedral angle between two l.s. planes) are estimated using the full covariance matrix. The cell esds are taken into account individually in the estimation of esds in distances, angles and torsion angles; correlations between esds in cell parameters are only used when they are defined by crystal symmetry. An approximate (isotropic) treatment of cell esds is used for estimating esds involving l.s. planes.
Refinement. Refinement of F^2^ against ALL reflections. The weighted R-factor wR and goodness of fit S are based on F^2^, conventional R-factors R are based on F, with F set to zero for negative F^2^. The threshold expression of F^2^ > 2sigma(F^2^) is used only for calculating R-factors(gt) etc. and is not relevant to the choice of reflections for refinement. R-factors based on F^2^ are statistically about twice as large as those based on F, and R- factors based on ALL data will be even larger.

### Fractional atomic coordinates and isotropic or equivalent isotropic displacement parameters (Å^2^)

**Table:**

	*x*	*y*	*z*	*U*_iso_*/*U*_eq_	Occ. (<1)
S1	0.29327 (3)	0.31834 (4)	−0.0279 (2)	0.0491 (2)	
O1	0.10485 (13)	0.45699 (12)	0.4998 (7)	0.0729 (8)	
H1	0.139 (2)	0.454 (3)	0.396 (12)	0.13 (2)*	
O2A	0.2600 (4)	0.6160 (5)	−0.080 (3)	0.086 (2)	0.70
O2B	0.2404 (9)	0.6148 (14)	−0.184 (7)	0.113 (9)	0.30
O3	0.17722 (11)	0.54989 (12)	0.0569 (8)	0.0883 (10)	
N1	0.19752 (11)	0.39116 (14)	0.2458 (6)	0.0487 (6)	
N2	0.43249 (11)	0.42497 (15)	−0.4324 (6)	0.0524 (7)	
C1	0.10020 (15)	0.38418 (19)	0.6356 (8)	0.0537 (8)	
C2	0.05554 (16)	0.3758 (2)	0.8360 (8)	0.0656 (10)	
H2	0.0303	0.4203	0.8765	0.079*	
C3	0.04824 (15)	0.3022 (2)	0.9757 (10)	0.0681 (9)	
H3	0.0176	0.2972	1.1080	0.082*	
C4	0.08544 (16)	0.2361 (2)	0.9225 (8)	0.0649 (9)	
H4	0.0803	0.1867	1.0189	0.078*	
C5	0.13008 (15)	0.2433 (2)	0.7273 (8)	0.0568 (8)	
H5	0.1553	0.1984	0.6919	0.068*	
C6	0.13862 (13)	0.31758 (16)	0.5781 (7)	0.0459 (7)	
C7	0.18721 (14)	0.32333 (19)	0.3779 (7)	0.0496 (8)	
H7	0.2133 (13)	0.2791 (19)	0.362 (7)	0.058 (10)*	
C8	0.24460 (12)	0.39946 (16)	0.0600 (7)	0.0449 (7)	
C9	0.26209 (12)	0.47141 (15)	−0.0714 (8)	0.0453 (7)	
C10	0.31599 (13)	0.46149 (16)	−0.2347 (7)	0.0458 (7)	
C11	0.34991 (14)	0.52626 (18)	−0.3940 (8)	0.0577 (9)	
H11A	0.3687	0.5645	−0.2645	0.069*	
H11B	0.3216	0.5573	−0.5081	0.069*	
C12	0.39840 (14)	0.48860 (19)	−0.5785 (7)	0.0569 (8)	
H12A	0.3795	0.4647	−0.7420	0.068*	
H12B	0.4260	0.5317	−0.6400	0.068*	
C13	0.39395 (13)	0.35399 (18)	−0.3697 (7)	0.0517 (8)	
H13A	0.4153	0.3161	−0.2474	0.062*	
H13B	0.3841	0.3248	−0.5402	0.062*	
C14	0.33712 (13)	0.38294 (17)	−0.2328 (7)	0.0468 (7)	
C15	0.48474 (16)	0.3986 (2)	−0.5965 (8)	0.0727 (11)	
H15A	0.5112	0.4449	−0.6272	0.109*	
H15B	0.4711	0.3773	−0.7722	0.109*	
H15C	0.5063	0.3561	−0.4979	0.109*	
C16	0.23215 (14)	0.55284 (17)	−0.0432 (10)	0.0571 (8)	
C17	0.1444 (2)	0.6277 (2)	0.0843 (15)	0.1079 (19)	
H17A	0.1400	0.6413	0.2799	0.129*	
H17B	0.1674	0.6716	−0.0041	0.129*	
C18	0.0884 (2)	0.6222 (3)	−0.036 (2)	0.166 (4)	
H18A	0.0663	0.6724	−0.0026	0.249*	
H18B	0.0666	0.5764	0.0434	0.249*	
H18C	0.0928	0.6139	−0.2325	0.249*	

### Atomic displacement parameters (Å^2^)

**Table:**

	*U*^11^	*U*^22^	*U*^33^	*U*^12^	*U*^13^	*U*^23^
S1	0.0510 (4)	0.0387 (3)	0.0577 (5)	−0.0018 (3)	−0.0006 (4)	0.0037 (4)
O1	0.0915 (18)	0.0470 (12)	0.080 (2)	0.0143 (11)	0.0188 (17)	0.0050 (14)
O2B	0.071 (12)	0.067 (9)	0.20 (3)	0.008 (8)	0.023 (12)	0.057 (13)
O2A	0.074 (5)	0.034 (2)	0.150 (8)	−0.008 (3)	0.006 (5)	0.000 (4)
O3	0.0702 (15)	0.0412 (11)	0.153 (3)	0.0134 (10)	0.0125 (18)	0.0042 (17)
N1	0.0494 (13)	0.0428 (13)	0.0538 (17)	−0.0065 (10)	−0.0035 (13)	−0.0020 (12)
N2	0.0510 (14)	0.0611 (15)	0.0451 (16)	−0.0100 (12)	−0.0020 (12)	−0.0006 (13)
C1	0.0553 (18)	0.0520 (17)	0.054 (2)	−0.0013 (14)	−0.0023 (16)	−0.0028 (16)
C2	0.068 (2)	0.059 (2)	0.070 (3)	0.0040 (16)	0.0080 (19)	−0.0106 (19)
C3	0.0651 (18)	0.078 (2)	0.061 (2)	−0.0085 (17)	0.014 (2)	−0.001 (2)
C4	0.072 (2)	0.0601 (18)	0.062 (3)	−0.0068 (16)	0.006 (2)	0.0011 (19)
C5	0.0626 (19)	0.0486 (17)	0.059 (2)	−0.0020 (15)	0.0021 (18)	−0.0007 (16)
C6	0.0500 (16)	0.0436 (15)	0.0440 (17)	−0.0025 (12)	−0.0052 (14)	−0.0033 (14)
C7	0.0480 (16)	0.0423 (16)	0.059 (2)	−0.0010 (13)	−0.0046 (14)	−0.0015 (15)
C8	0.0411 (14)	0.0402 (14)	0.053 (2)	−0.0044 (11)	−0.0078 (13)	−0.0001 (14)
C9	0.0470 (14)	0.0384 (13)	0.050 (2)	−0.0062 (11)	−0.0098 (16)	−0.0007 (15)
C10	0.0487 (15)	0.0394 (15)	0.0494 (19)	−0.0056 (12)	−0.0098 (16)	−0.0005 (14)
C11	0.0621 (19)	0.0505 (17)	0.061 (2)	−0.0111 (15)	−0.0065 (18)	0.0088 (17)
C12	0.0663 (19)	0.0605 (17)	0.044 (2)	−0.0129 (15)	−0.0008 (17)	0.0017 (17)
C13	0.0527 (17)	0.0532 (16)	0.049 (2)	−0.0044 (14)	−0.0063 (15)	0.0006 (16)
C14	0.0450 (15)	0.0449 (16)	0.051 (2)	−0.0069 (12)	−0.0093 (15)	0.0025 (15)
C15	0.065 (2)	0.092 (3)	0.061 (3)	−0.0069 (18)	0.0067 (19)	−0.001 (2)
C16	0.0576 (17)	0.0414 (15)	0.072 (2)	−0.0035 (13)	−0.005 (2)	0.001 (2)
C17	0.095 (3)	0.0472 (19)	0.182 (6)	0.025 (2)	0.000 (4)	−0.009 (3)
C18	0.108 (4)	0.109 (4)	0.280 (10)	0.057 (3)	−0.054 (6)	−0.071 (6)

### Geometric parameters (Å, º)

**Table:**

S1—C8	1.745 (3)	C9—C16	1.478 (4)
S1—C14	1.730 (3)	C10—C11	1.497 (4)
O1—H1	0.91 (5)	C11—C12	1.515 (5)
O2A—O2B	0.66 (3)	C11—H11A	0.9700
O3—C17	1.455 (4)	C11—H11B	0.9700
N1—C8	1.375 (4)	C12—H12A	0.9700
N2—C12	1.454 (4)	C12—H12B	0.9700
N2—C15	1.459 (4)	C13—N2	1.458 (4)
C1—O1	1.349 (4)	C13—C14	1.489 (4)
C2—C1	1.383 (5)	C13—H13A	0.9700
C2—H2	0.9300	C13—H13B	0.9700
C3—C2	1.375 (5)	C14—C10	1.352 (4)
C3—C4	1.370 (4)	C15—H15A	0.9600
C3—H3	0.9300	C15—H15B	0.9600
C4—C5	1.364 (5)	C15—H15C	0.9600
C4—H4	0.9300	C16—O2A	1.203 (10)
C5—C6	1.411 (4)	C16—O2B	1.22 (3)
C5—H5	0.9300	C16—O3	1.303 (4)
C6—C1	1.396 (4)	C17—C18	1.366 (7)
C7—N1	1.287 (4)	C17—H17A	0.9700
C7—C6	1.442 (4)	C17—H17B	0.9700
C7—H7	0.92 (3)	C18—H18A	0.9600
C8—C9	1.378 (4)	C18—H18B	0.9600
C9—C10	1.432 (4)	C18—H18C	0.9600
			
C14—S1—C8	91.59 (14)	C12—C11—H11B	109.3
C1—O1—H1	106 (3)	H11A—C11—H11B	107.9
C16—O3—C17	117.6 (3)	N2—C12—C11	111.4 (3)
C7—N1—C8	122.5 (3)	N2—C12—H12A	109.3
C12—N2—C13	110.8 (2)	N2—C12—H12B	109.3
C12—N2—C15	110.7 (3)	C11—C12—H12A	109.3
C15—N2—C13	109.9 (3)	C11—C12—H12B	109.3
O1—C1—C2	118.4 (3)	H12A—C12—H12B	108.0
O1—C1—C6	122.0 (3)	N2—C13—C14	109.5 (2)
C2—C1—C6	119.6 (3)	N2—C13—H13A	109.8
C1—C2—H2	119.7	N2—C13—H13B	109.8
C3—C2—C1	120.5 (3)	C14—C13—H13A	109.8
C3—C2—H2	119.7	C14—C13—H13B	109.8
C2—C3—H3	119.6	H13A—C13—H13B	108.2
C4—C3—C2	120.9 (4)	C10—C14—S1	112.2 (2)
C4—C3—H3	119.6	C10—C14—C13	125.5 (3)
C3—C4—H4	120.2	C13—C14—S1	122.1 (2)
C5—C4—C3	119.6 (3)	N2—C15—H15A	109.5
C5—C4—H4	120.2	N2—C15—H15B	109.5
C4—C5—C6	121.2 (3)	N2—C15—H15C	109.5
C4—C5—H5	119.4	H15A—C15—H15B	109.5
C6—C5—H5	119.4	H15A—C15—H15C	109.5
C1—C6—C5	118.3 (3)	H15B—C15—H15C	109.5
C1—C6—C7	122.1 (3)	O2A—C16—O3	123.9 (5)
C5—C6—C7	119.5 (3)	O2A—C16—C9	120.9 (5)
N1—C7—C6	121.0 (3)	O2B—C16—O3	111.8 (11)
N1—C7—H7	121 (2)	O2B—C16—C9	127.8 (14)
C6—C7—H7	118 (2)	O3—C16—C9	114.6 (3)
N1—C8—S1	123.2 (2)	C18—C17—O3	110.8 (4)
N1—C8—C9	126.2 (3)	C18—C17—H17A	109.5
C9—C8—S1	110.5 (2)	C18—C17—H17B	109.5
C8—C9—C10	112.9 (2)	O3—C17—H17A	109.5
C8—C9—C16	125.8 (3)	O3—C17—H17B	109.5
C10—C9—C16	121.3 (3)	H17A—C17—H17B	108.1
C9—C10—C11	128.0 (3)	C17—C18—H18A	109.5
C14—C10—C9	112.8 (3)	C17—C18—H18B	109.5
C14—C10—C11	119.2 (3)	C17—C18—H18C	109.5
C10—C11—C12	111.7 (2)	H18A—C18—H18B	109.5
C10—C11—H11A	109.3	H18A—C18—H18C	109.5
C10—C11—H11B	109.3	H18B—C18—H18C	109.5
C12—C11—H11A	109.3		
			
C14—S1—C8—N1	−175.9 (3)	C8—C9—C10—C14	2.1 (4)
C14—S1—C8—C9	1.3 (3)	C16—C9—C10—C11	0.3 (5)
C8—S1—C14—C10	−0.1 (3)	C16—C9—C10—C14	179.2 (3)
C8—S1—C14—C13	175.6 (3)	C8—C9—C16—O2A	153.8 (7)
C16—O3—C17—C18	129.0 (6)	C8—C9—C16—O2B	−168.6 (14)
C7—N1—C8—S1	2.8 (4)	C8—C9—C16—O3	−17.9 (5)
C7—N1—C8—C9	−173.9 (3)	C10—C9—C16—O2A	−22.9 (8)
C13—N2—C12—C11	66.6 (3)	C10—C9—C16—O2B	14.7 (14)
C15—N2—C12—C11	−171.1 (3)	C10—C9—C16—O3	165.4 (3)
C3—C2—C1—O1	178.6 (4)	C9—C10—C11—C12	−170.4 (3)
C3—C2—C1—C6	−0.9 (5)	C14—C10—C11—C12	10.8 (4)
C4—C3—C2—C1	0.9 (6)	C10—C11—C12—N2	−44.3 (3)
C2—C3—C4—C5	−0.4 (6)	C14—C13—N2—C12	−50.4 (4)
C3—C4—C5—C6	−0.2 (5)	C14—C13—N2—C15	−173.1 (3)
C4—C5—C6—C1	0.2 (5)	N2—C13—C14—S1	−157.9 (2)
C4—C5—C6—C7	178.6 (3)	N2—C13—C14—C10	17.1 (4)
C5—C6—C1—O1	−179.1 (3)	S1—C14—C10—C9	−1.1 (4)
C5—C6—C1—C2	0.4 (5)	S1—C14—C10—C11	177.9 (2)
C7—C6—C1—O1	2.5 (5)	C13—C14—C10—C9	−176.5 (3)
C7—C6—C1—C2	−178.0 (3)	C13—C14—C10—C11	2.4 (5)
C6—C7—N1—C8	178.0 (3)	O3—C16—O2A—O2B	−76 (4)
N1—C7—C6—C1	0.9 (5)	C9—C16—O2A—O2B	113 (3)
N1—C7—C6—C5	−177.5 (3)	O3—C16—O2B—O2A	120 (3)
S1—C8—C9—C10	−2.1 (3)	C9—C16—O2B—O2A	−89 (4)
S1—C8—C9—C16	−179.1 (3)	O2A—C16—O3—C17	10.0 (9)
N1—C8—C9—C10	175.0 (3)	O2B—C16—O3—C17	−23.2 (16)
N1—C8—C9—C16	−2.0 (5)	C9—C16—O3—C17	−178.6 (4)
C8—C9—C10—C11	−176.8 (3)		

### Hydrogen-bond geometry (Å, º)

Cg1 is the centroid of ring *B* (S1/C8–C10/C14).

**Table:**

*D*—H···*A*	*D*—H	H···*A*	*D*···*A*	*D*—H···*A*
O1—H1···O3	0.90 (5)	2.40 (5)	3.053 (4)	129 (4)
O1—H1···N1	0.90 (5)	1.79 (5)	2.605 (4)	148 (4)
C12—H12*A*···*Cg*1^i^	0.97	2.77	3.701 (3)	161

Symmetry code: (i) *x*, *y*, *z*−1.
